# Potato Virus Y Coat Protein Interacts With Cytosolic Phosphoglucomutase and Reprogrammes Host Primary Metabolism During Disease Development

**DOI:** 10.1111/mpp.70368

**Published:** 2026-11-09

**Authors:** Prabu Gnanasekaran, Ying Zhai, Hanu R. Pappu

**Affiliations:** ^1^ Department of Plant Pathology Washington State University Pullman Washington USA

**Keywords:** host primary metabolism, nucleic acid synthesis, phosphoglucomutase, potato virus Y, viral replication

## Abstract

Potato virus Y (PVY) is one of the top five economically important plant viruses in the world that affects several plant species including potato, pepper and tomato. We have combined untargeted metabolomics with protein–protein interaction studies to understand the mechanistic details of PVY‐induced metabolic changes that lead to disease development. For this purpose, PVY necrotic strain, PVY^NTN^, and *Nicotiana benthamiana* were used as a model system. Gas chromatography–mass spectrometry (GC–MS)‐based metabolomic profiling revealed that PVY infection significantly alters carbohydrate and amino acid metabolism, with elevated levels of glucose‐6‐phosphate, fructose‐6‐phosphate and key amino acids associated with nucleotide biosynthesis. Using yeast two‐hybrid, bimolecular fluorescence complementation and GST pull‐down assays, we identified a direct interaction between the PVY coat protein (CP) and cytosolic phosphoglucomutase (cPGM), a central enzyme regulating cytosolic sugar‐phosphate partitioning among different pathways. Subcellular localization analyses showed that PVY CP localizes to the cytosol, forms aggregates and alters cPGM distribution, recruiting it into CP‐associated cytosolic aggregates. Silencing of *cPGM* restricted PVY accumulation and disease development, indicating that cPGM is required for efficient PVY infection and contributes to disease development in *N. benthamiana*. Based on these findings, we propose a working model in which PVY CP hijacks cPGM to redirect carbohydrate flux towards respiratory and pentose phosphate pathways, thereby increasing the availability of energy, reducing equivalents, and biosynthetic precursors required for viral replication. Together, this study provides mechanistic insight into PVY‐induced metabolic reprogramming and identifies cPGM as a key host factor linking viral infection to primary metabolism.

## Graphical abstract

Potato virus Y (PVY) coat protein (CP) interacts with cytosolic phosphoglucomutase (cPGM) in the cytosol, redirecting carbohydrate flux towards respiratory and pentose phosphate pathways. Consequently, the plant cell can meet the increased ATP, NADPH and biosynthetic precursor requirements for viral RNA synthesis, thereby promoting PVY accumulation and disease development.

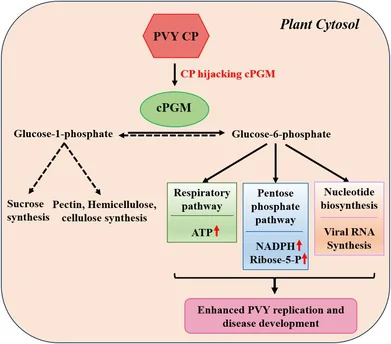

## 1 Introduction

Plants respond to viruses with multilayered defence strategies that primarily include innate immune responses, RNA silencing, resistance (R) gene‐mediated immunity, programmed cell death, ubiquitin‐mediated viral protein degradation and altered metabolism. Among them, metabolic changes are mediated through complex plant–virus interactions that are often difficult to understand and unique for a particular host–virus system (Mandadi and Scholthof 2013; Wu et al. 2019). On the other hand, being obligate intracellular parasites, viruses exploit the host cellular machinery throughout their infection cycle for their own replication, movement and pathogenesis. Viruses modulate the highly orchestrated cellular events by interacting with various host factors to favour enhanced virus replication and pathogenesis. Viruses counteract host immune response through interaction with specific host factors and manipulate host cellular pathways (Wu et al. 2019; Lewsey et al. 2018). Thus, both plant and virus contribute to the cellular reprogramming of plant cells that occur during plant–virus interactions. Such massive reprogramming is associated with increased demand for energy, reducing equivalents and carbon skeletons that are provided by primary metabolic pathways.

Key carbohydrate metabolic pathways in plant cells include photosynthesis, cellular respiratory pathways such as glycolysis and the tricarboxylic acid (TCA) cycle, and the pentose phosphate pathway, an alternative glucose catabolic pathway that generates NADPH and provides ribose sugars required for nucleic acid synthesis. Photosynthesis is the process of conversion of carbon dioxide into glucose that occurs in chloroplasts and has two phases: light‐dependent reaction and Calvin cycle. The glucose produced by photosynthesis is the ultimate source for generating energy and synthetic precursors required for the cell. The excess glucose produced during active photosynthesis is used for starch and sucrose synthesis in chloroplasts and cytosol, respectively. In the dark, the direction of metabolic flow changes and these stored carbohydrates are mobilized and metabolized by glycolysis. Different carbohydrate metabolic pathways in plants overlap, and the transporters in the membrane of subcellular organelles such as chloroplasts and mitochondria mediate the movement of carbohydrate intermediates as sugar phosphates, including glucose‐1‐phosphate (Eveland and Jackson 2011; Lehninger et al. 2000).

Phosphoglucomutase (PGM) is an enzyme that catalyses the reversible conversion of glucose‐6‐phosphate to glucose‐1‐phosphate, and it exists as plastidial (pPGM) and cytosolic (cPGM) isoforms (Fettke et al. 2010). In chloroplasts, pPGM catalyses the reversible conversion of glucose‐6‐phosphate to glucose‐1‐phosphate. Glucose‐1‐phosphate in chloroplasts acts as a substrate for ADP‐glucose pyrophosphorylase, which catalyses the conversion of glucose‐1‐phosphate to ADP‐glucose, that acts as a precursor for starch synthesis. In the cytosol, cPGM is involved in controlling the partitioning of both sugar‐phosphate including glucose‐6‐phosphate and glucose‐1‐phosphate. Glucose‐6‐phosphate is primarily involved in respiratory pathways and the alternate pathway, pentose phosphate pathway. The pentose phosphate pathway provides ribose‐5‐phosphate and NADPH that are essential for RNA/DNA synthesis. Glucose‐1‐phosphate acts as a substrate for UDP‐glucose pyrophosphorylase, which catalyses the conversion of glucose‐1‐phosphate to UDP‐glucose, which acts as a precursor for synthesis of cellulose, hemicellulose and pectin and sucrose (Stitt and Zeeman 2012; Flütsch et al. 2022).

Viruses, throughout their infection cycle, require energy and building blocks for their nucleic acid and protein synthesis. Controlling the metabolic resources of the host is fundamental during host–pathogen interactions. Viruses have evolved extensively to manipulate multiple host cellular processes and modulate the metabolic pathways. Virus control of the host's metabolic resources is a complex process but has been better studied in animal virus systems compared to plant virus systems. Research on different animal virus systems has established that viruses manipulate the host metabolic pathways to divert energy and biosynthetic precursors towards virus replication and assembly. Upon infection, different animal viruses upregulate glycolysis, lipid biosynthesis and nucleotide metabolism to supply ATP and carbon skeletons required for virion production and assembly, turning infected cells into virus factories. Several studies on human viruses, including DNA viruses such as adenovirus, Epstein–Barr virus, human cytomegalovirus (HCMV), human papillomavirus (HPV) and Kaposi's sarcoma‐associated herpesvirus (KSHV) (Yogev et al. 2014; Munger et al. 2008; Thai et al. 2014; Xiao et al. 2014), as well as RNA viruses such as dengue, hepatitis C (HCV) and influenza A (Fontaine et al. 2015; Diamond et al. 2010; Ritter et al. 2010), have been reported to activate glycolytic flux. Such metabolic hijacking by viruses activates glucose and glutamine metabolism, provides energy and reducing equivalents such as ATP, NADH and NADPH, and supports virus multiplication (Goodwin et al. 2015).

Plant viruses are increasingly recognized for their ability to manipulate host primary metabolism to support infection. Brome mosaic virus (BMV), cucumber mosaic virus (CMV), sugarcane mosaic virus (SCMV), tobacco mosaic virus (TMV), tobacco rattle virus (TRV) and zucchini yellow mosaic virus (ZYMV) are reported to alter carbohydrate and amino acid metabolism, suggesting that reprogramming of central carbon metabolism is a common feature of plant virus infection (Shalitin and Wolf 2000; Jiang et al. 2022; López‐Gresa et al. 2012; Xu et al. 2008; Blua et al. 1994). Recently, SCMV was shown to recruit a glycolytic enzyme to viral replication complexes, providing direct evidence that plant viruses can exploit host metabolic enzymes to facilitate replication (Jiang et al. 2022). However, the mechanistic details of metabolic reprogramming and the role of cPGM and its potential targeting by viral proteins have not been explored.

Potato virus Y (PVY, *Potyvirus yituberosi*) is a single‐stranded, positive‐sense RNA virus and the type member of the genus *Potyvirus*, family *Potyviridae* (Gadhave et al. 2020; Inoue‐Nagata et al. 2022). PVY is ranked among the top five economically important plant viruses (Scholthof et al. 2011). PVY has a wide host range, infecting more than 150 plant species in 12 families, including potato, pepper and tomato (Kerlan and Moury 2008) and is transmitted by at least 50 different aphid species (Sigvald 1987, 1990). The virus contains a genome of about 9.7 kb and encodes a single polyprotein that is post‐translationally cleaved into P1‐pro, HC‐pro, P3, CI, VPg, NIa‐pro, NIb and coat protein (CP). A small open reading frame (ORF), termed PIPO (pretty interesting potyvirus ORF), is predicted to encode a ∼7 kDa protein and is embedded within the P3 coding region (Gadhave et al. 2020; Dougherty and Carrington 1988).

PVY is a complex biological entity that seems to evolve and generate new strains that are biologically distinct (Karasev and Gray 2013; Karasev et al. 2011). These include ordinary strain (PVY^O^), which induces mosaic symptoms on foliage, and tuber necrotic strain (PVY^N^), which causes necrotic ringspot disease in potato tuber of most cultivars (Gray 2007). Newer strains continue to emerge mostly through genetic recombination of existing strains (Gray 2007; Lorenzen et al. 2006; Green et al. 2017). A shift in the prevalence of original strains has been observed over the years in both Europe and the United States, with the recombinant necrotic strains PVY^NTN^ and PVY^N‐Wi^ being most prevalent in the United States, while PVY^NTN^ predominates in Europe (Blanchard et al. 2008; Kamangar et al. 2014). The use of certified virus‐free seed, managing vector populations and PVY‐resistant cultivars are the currently available virus management strategies (Karasev and Gray 2013; Paluchowska et al. 2024). PVY and its aphid vector have a wide host range that includes both crops and weeds. The highly prevalent necrotic strains often show only mild foliar symptoms and therefore go unnoticed during field inspections conducted for seed certification, likely contributing to their increased incidence (Gadhave et al. 2020).

In this study, we investigated the host metabolic changes that occur during the PVY necrotic strain (PVY^NTN^) infection in *Nicotiana benthamiana* plants and showed that a key host metabolic enzyme is targeted by PVY. Our findings from untargeted metabolomic profiling indicate that PVY reprogrammes host carbohydrate metabolism towards glycolytic and pentose phosphate pathways. Increased levels of glucose‐6‐phosphate and glutamine in the infected plants suggest that, similar to human viruses, PVY also hijacks host metabolism, activates glucose and glutamine metabolism, provides energy and reducing equivalents, and supports virus multiplication. Using a yeast two‐hybrid (Y2H) approach, we have identified and confirmed interaction between PVY‐CP protein and cytosolic phosphoglucomutase (cPGM), a key enzyme that regulates the carbohydrate partitioning in the cytosol of the host. Silencing of *cPGM* restricted virus accumulation and disease development. Together, these findings suggest that the PVY coat protein–cPGM interaction plays an important role in virus‐induced metabolic reprogramming.

## 2 Results

### 2.1 PVY Infection Modulates Carbohydrate Metabolism

Changes in leaf metabolome in PVY‐infected plants were determined using gas chromatography–mass spectrometry (GC–MS). For this purpose, *N. benthamiana* plants were mechanically inoculated with a PVY^NTN^ strain (GenBank accession no. ON866943). No symptoms were observed in the mock‐inoculated *N. benthamiana* plants, while PVY^NTN^‐inoculated plants showed symptoms such as leaf curling, chlorotic mosaic and stunted growth (Figure 1A). Leaf samples from PVY^NTN^‐ and mock‐infected plants were collected at 14 days post‐inoculation (dpi), and a non‐targeted metabolomic analysis was performed. In response to PVY infection, many of the metabolites related to carbohydrate metabolism were found to be differentially regulated in comparison to leaves of mock‐infected plants (Figure 1B).

**FIGURE 1:**
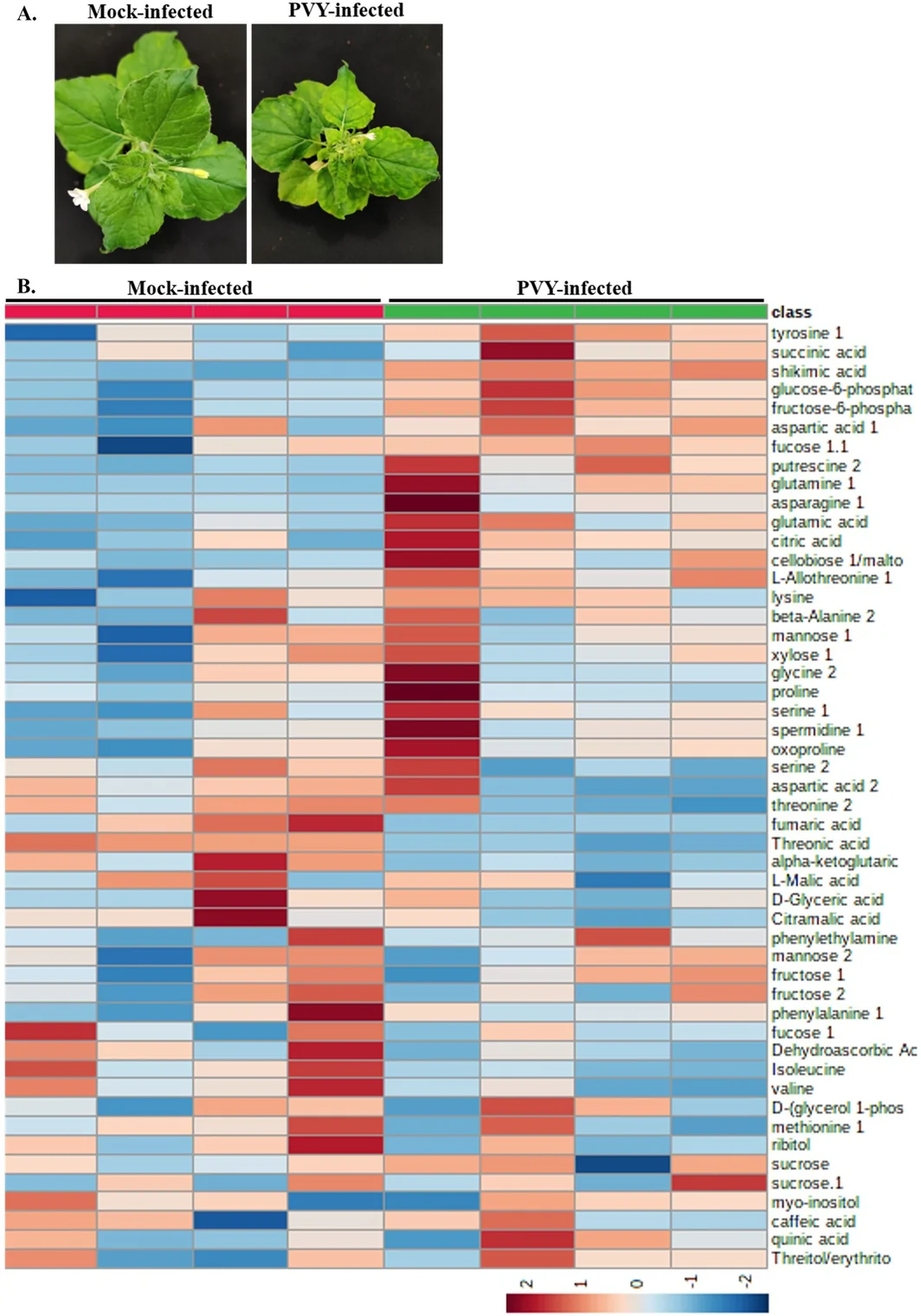
Potato virus Y (PVY) infection modulates host primary metabolism in *Nicotiana benthamiana*. (A) Mock‐ and PVY tuber necrotic strain (PVY^NTN^)‐inoculated *N. benthamiana* plants at 14 days post‐inoculation (dpi). (B) Heatmap depicting the differentially altered metabolites in the leaves of the mock‐ and PVY‐infected *N. benthamiana* plants at 14 dpi. Metabolic changes in the leaves of the mock‐ and PVY‐infected *N. benthamiana* plants were analysed by untargeted gas chromatography–mass spectrometry (GC–MS) with four biological replicates. The heatmap was generated using MetaboAnalyst v. 5.0 and based on normalized metabolite intensities, showing the altered metabolites in the leaves of the mock‐ and PVY‐infected *N. benthamiana* plants at 14 dpi. The blue‐to‐red colour scale bar represents −2 to +2 log fold changes in the metabolite abundance.

Fold‐change analysis was performed. Several metabolites, including fructose‐6‐phosphate, glucose‐6‐phosphate, citric acid, glutamine, glycine and sucrose, showed increased abundance in PVY‐infected leaves compared to mock‐inoculated plants (Figure 2). In contrast, metabolites such as α‐ketoglutaric acid, fumaric acid, isoleucine, threonine and valine were reduced (Figure 2). To understand the primary target of the pathways that are affected by PVY, a list of metabolites that showed at least 1.5‐fold change during PVY infection was selected. The levels of asparagine, maltose/cellobiose, citric acid, fructose‐6‐phosphate, glucose‐6‐phosphate, glutamine, proline, putrescine, shikimic acid, spermidine and tyrosine metabolite were found to be elevated in the leaves of infected plants in comparison to those of mock‐inoculated plants (Figure S1), while the relative levels of α‐ketoglutaric acid, aspartic acid, fumaric acid, isoleucine, threonic acid, threonine and valine were found to be reduced in infected leaves compared to those of mock‐inoculated plants (Figure S2). Results of the pathway analysis indicated that PVY infection primarily targets starch and sucrose metabolism with altered levels of seven key intermediates of this pathway that include carbohydrate metabolism, affecting starch and sucrose metabolism, TCA cycle and with altered levels of several amino acid metabolic pathways, including altered arginine biosynthesis; alanine, aspartate, glutamate metabolism; glutathione metabolism; glycine, serine, threonine metabolism; and valine, leucine and isoleucine biosynthesis. The elevated levels of fructose‐6‐phosphate, glucose‐6‐phosphate and citric acid in the PVY‐infected plants indicate altered carbohydrate metabolism, with energy flux likely redirected towards cellular respiratory pathways. Altered levels of amino acid metabolites further suggest a coupling between carbohydrate metabolism and amino acid biosynthesis, potentially reflecting the change in carbon flux from glycolysis towards the TCA cycle to support energy production and biosynthetic demands during PVY infection. Taken together, these results suggest that PVY infection is associated with alterations in primary metabolism, particularly in carbohydrate and amino acid pathways, which may contribute to the metabolic environment supporting viral infection. Metabolites associated with carbohydrate metabolism, the TCA cycle and amino acid biosynthesis were among the most significantly altered classes.

**FIGURE 2:**
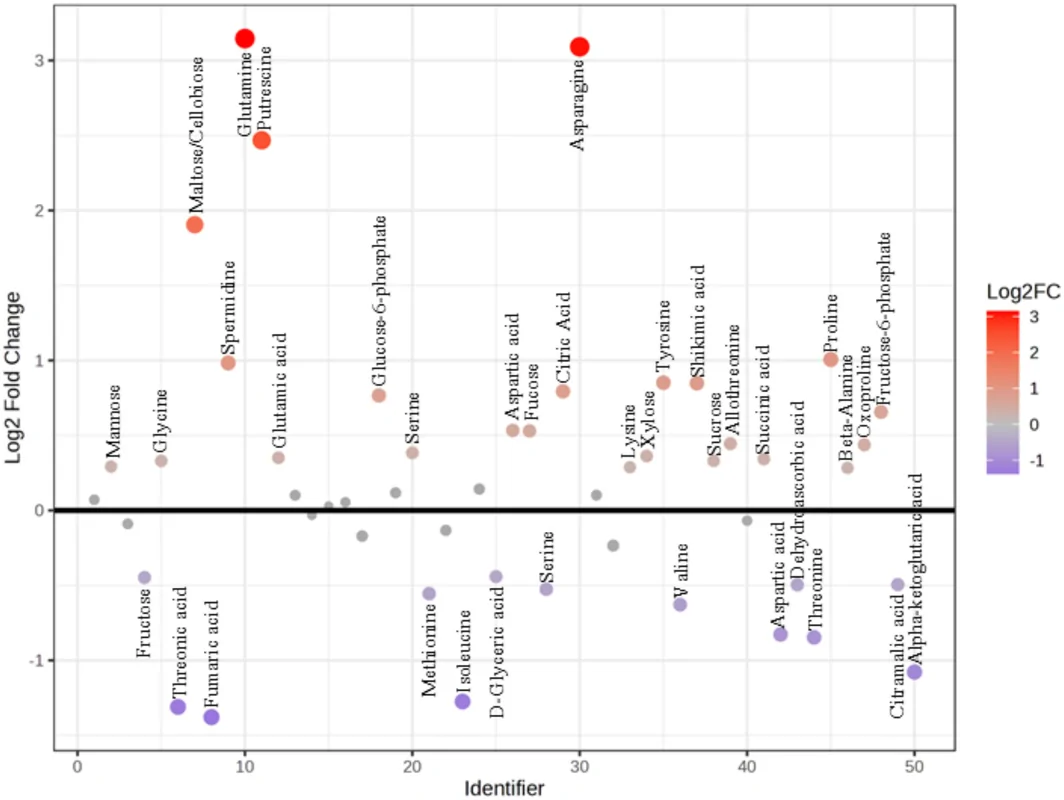
Fold change analysis of metabolites altered during potato virus Y (PVY) infection. Fold change representation of differentially altered metabolites in the PVY‐ versus mock‐infected *Nicotiana benthamiana* leaf samples. Fold change analysis was performed using MetaboAnalyst v. 5.0 and data are represented as log_2_ fold change. Fold changes were calculated based on normalized metabolite intensities, with mock‐infected samples used as the reference. Differentially altered metabolites with increased or decreased abundance in PVY‐infected samples are represented with red and blue colour, respectively.

### 2.2 PVY Interacts With Cytosolic Phosphoglucomutase

Given the prominent changes in the primary metabolism, we next investigated whether PVY directly targets host factors involved in carbohydrate partitioning. To elucidate the molecular mechanism of how PVY targets the carbohydrate metabolism, *N. benthamiana* Y2H cDNA library was screened to identify the host protein(s) that interact with viral proteins (Gnanasekaran et al. 2023; Gnanasekaran and Pappu 2023c). We identified the potential interacting partners of coat protein (CP) by the *N. benthamiana* Y2H library screening (Table 1). *N. benthamiana* cytosolic phosphoglucomutase (cPGM) was found as one of the interacting partners with the CP. cPGM is an enzyme that catalyses the reversible interconversion of glucose‐1‐phosphate and glucose‐6‐phosphate in the cytosol of plant cells. cPGM plays a critical role in glucose metabolism by regulating the partitioning of glucose phosphates into different metabolic pathways, directing glucose‐1‐phosphate towards biosynthetic pathways such as sucrose, pectin and cellulose synthesis and glucose‐6‐phosphate towards respiratory pathways such as glycolysis and the pentose phosphate pathway (Fernie et al. 2002; Lytovchenko et al. 2002). The interaction between CP and cPGM was confirmed by Y2H assay. *Saccharomyces cerevisiae* Y2HGold co‐transformed with AD‐cPGM and BD‐CP was able to grow on SD−Leu−Trp−His selection plates with 1 mM 3‐amino‐1,2,4‐triazole (3‐AT), whereas yeast cells harbouring the negative control plasmid combinations, namely AD and BD, AD‐cPGM and BD, or BD‐CP and AD, did not grow (Figure 3). AD‐TAg and BD‐P53 acted as positive control. These observations confirmed the interaction between CP and cPGM in yeast cells (Figure 3).

**TABLE 1:** List of potential interacting partners^a^ of potato virus Y‐encoded coat protein identified through yeast two‐hybrid (Y2H) library screening.

	Coat protein‐interacting proteins
1.	Peptidyl‐prolyl cis‐trans isomerase FKBP16‐4
2.	Cytoplasmic phosphoglucomutase 1
3.	Plastid‐lipid associated protein PAP/fibrillin family protein
4.	Cysteine‐rich extensin‐like protein (PEARL1)
5.	Sucrose non‐fermenting 1‐related kinase
6.	Uncharacterized LOC109213019, transcript variant X1 protein (2 hits)
7.	Calcineurin B‐like proteins (CBL)‐interacting protein kinase 1
8.	Calcium‐dependent lipid‐binding (CaLB domain) family protein
9.	Chloroplastic phosphoglucomutase 1
10.	Chloroplastic‐like, phosphatidylglycerol/phosphatidylinositol transfer protein
11.	Plastocyanin A′/A″ protein
12.	Polyubiquitin protein

^a^ Interacting partners are listed in descending order according to the number of hits obtained in the Y2H screening.

**FIGURE 3:**
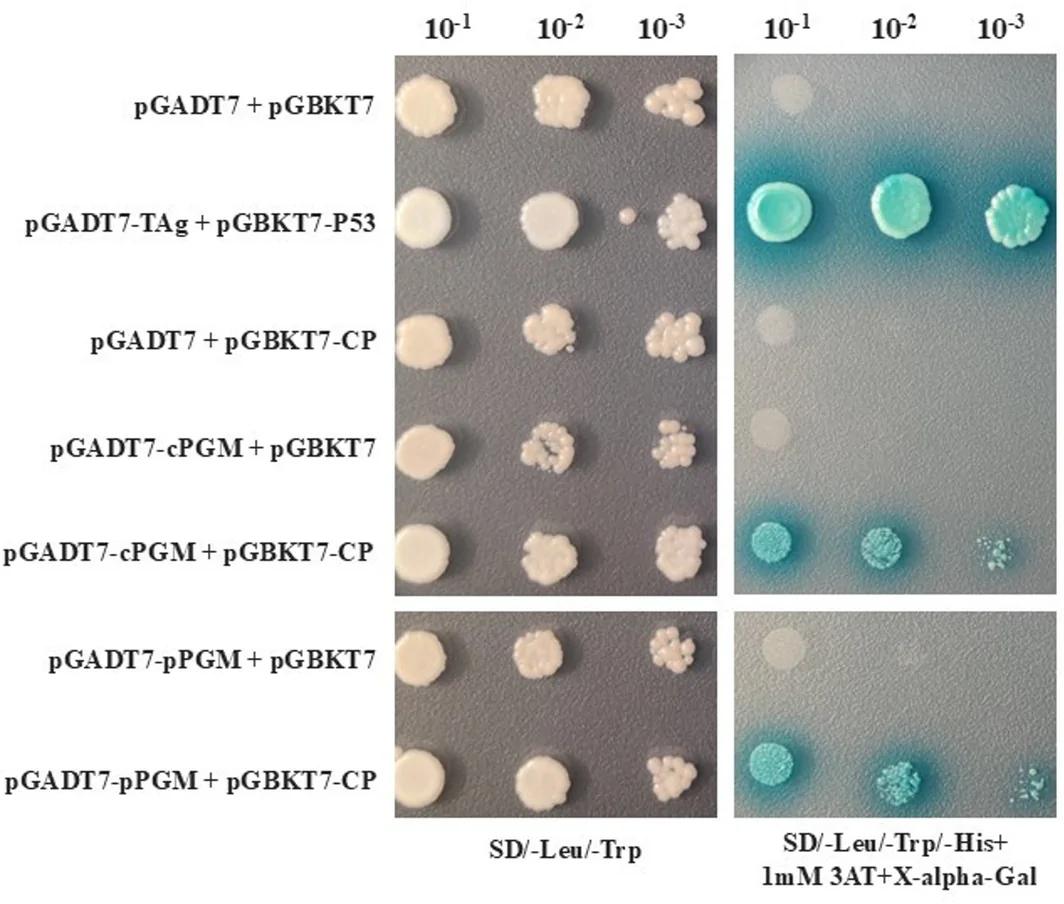
Potato virus Y‐encoded coat protein (CP) interacts with *Nicotiana benthamiana* cytosolic phosphoglucomutase (cPGM) in the yeast cell. Yeast two‐hybrid assay was performed using *Saccharomyces cerevisiae* Y2HGold. The different plasmid combinations pGADT7 + pGBKT7, pGADT7‐Tag + pGBKT7‐P53, pGADT7 + pGBKT7‐CP, pGADT7‐cPGM + pGBKT7, pGADT7‐cPGM + pGBKT7‐CP, pGADT7‐pPGM + pGBKT7 and pGADT7‐pPGM + pGBKT7‐CP were co‐transformed into Y2HGold cells. The co‐transformants were grown on either synthetic dropout (SD) medium lacking Leu and Trp (SD−Leu−Trp) or selection medium lacking Leu, Trp and His (SD−Leu−Trp−His) supplemented with 1 mM 3‐amino‐1,2,4‐triazole (3AT) and X‐α‐Gal as serial dilution from cultures adjusted to OD_600_ of 1.0. The pGADT7‐TAg + pGBKT7‐P53 and pGADT7 + pGBKT7 co‐transformants served as positive and negative controls, respectively.

The CP–cPGM interaction was also confirmed in planta by bimolecular fluorescence complementation (BiFC) assay. BiFC assays were carried out in *N. benthamiana* plants using the pSITE vectors. YFP fluorescence was observed in the epidermal cells of the leaves co‐infiltrated with *Agrobacterium tumefaciens* cells containing the combination of nYFP‐CP + cYFP‐cPGM. The reconstituted YFP fluorescence from CP–cPGM was detected predominantly in the cytoplasm (Figure 4A). Distinct CP‐associated cytoplasmic aggregates were also observed, as indicated by arrows. No reconstituted YFP fluorescence was detected in nYFP‐CP + cYFP‐pPGM‐infiltrated leaves, indicating no CP–pPGM interaction (Figure S3). No fluorescence was observed in the epidermal cells of leaves co‐infiltrated with negative control combinations nYFP + cYFP, nYFP‐CP + cYFP, nYFP + cYFP‐cPGM and nYFP + cYFP‐pPGM (Figure S3). Chloroplasts of the epidermal cells were detected by capturing chlorophyll autofluorescence. These results indicated that the PVY CP specifically interacts with cPGM, but not with pPGM and that the CP–cPGM interaction‐associated fluorescence was localized predominantly at the cell periphery and not in chloroplasts.

**FIGURE 4:**
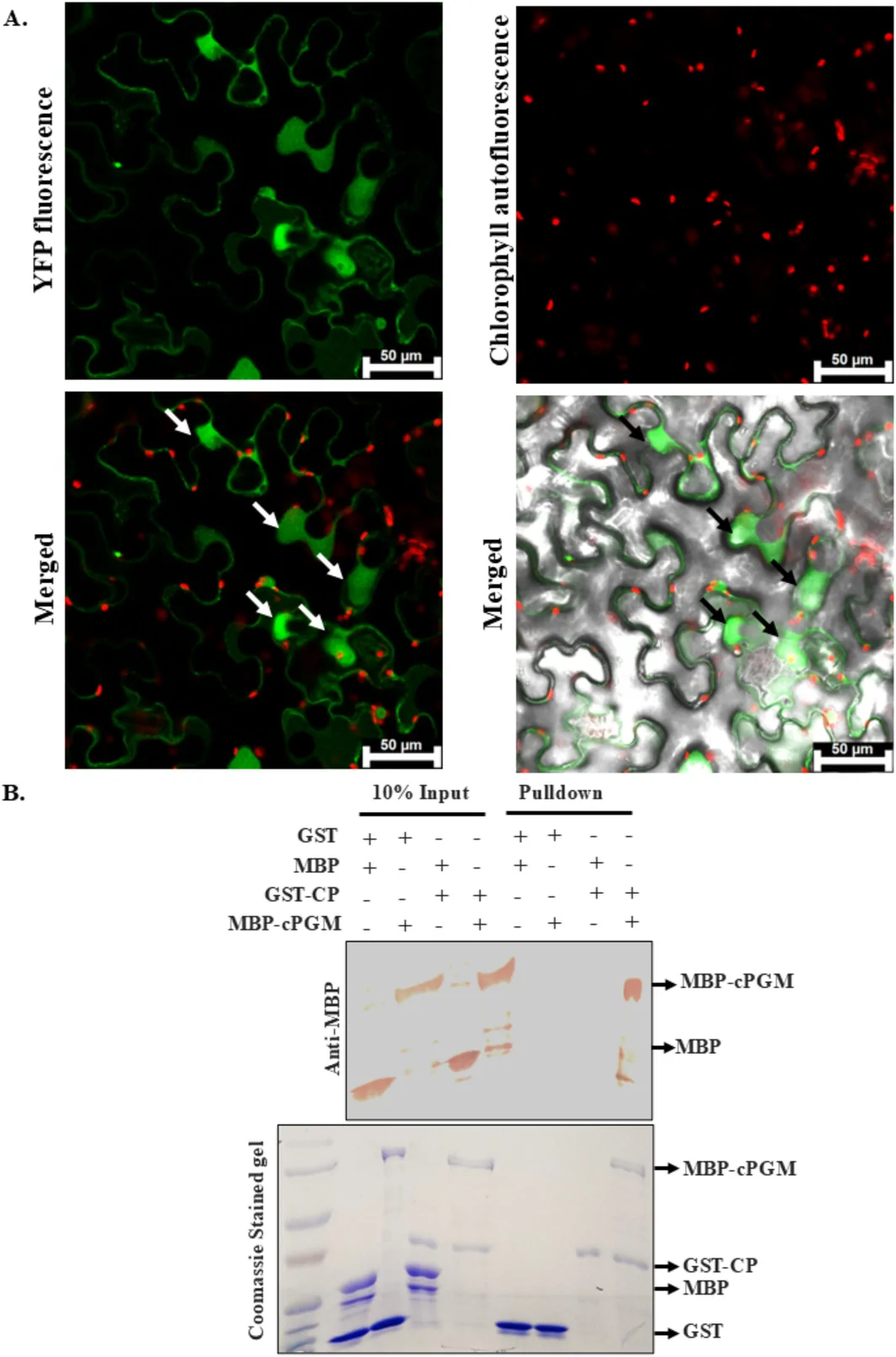
Potato virus Y‐encoded coat protein (CP) interacts with *Nicotiana benthamiana* cytosolic phosphoglucomutase (cPGM). (A) Bimolecular fluorescence complementation (BiFC) assays. *Agrobacterium tumefaciens* cells containing BiFC assay constructs were co‐infiltrated into the abaxial surface of leaves of 3‐week‐old *N. benthamiana* plants. The epidermal cells of the infiltrated leaves were visualized under a confocal microscope between 48 and 72 h post‐infiltration. Images show reconstituted YFP fluorescence, chlorophyll autofluorescence and merged channels in *N. benthamiana* leaves co‐infiltrated with nYFP‐CP and cYFP‐cPGM. Arrows indicate CP‐associated cytoplasmic aggregates. Scale bar represents 50 μm. (B) In vitro GST pull‐down assay. An equal amount purified GST/GST‐CP protein was used to pull down MBP/MBP‐cPGM protein. Immunoblotting was carried out using anti‐MBP antibodies. A representative Coomassie‐stained gel was used to monitor the amounts of protein present in the input and pull‐down fractions.

To further validate the CP–cPGM interaction, we performed a glutathione S‐transferase (GST) pull‐down assay. As the CP–pPGM interaction was not detected in the Y2H or BiFC assays and pPGM could not be purified, we focused on CP and cPGM. Bacterially expressed and purified GST, maltose‐binding protein (MBP), GST‐CP and MBP‐cPGM were used in the in vitro pull‐down assay. MBP‐cPGM was pulled down by GST‐CP but not by GST alone. In addition, MBP alone was not pulled down by either GST‐CP or GST. These results support a specific interaction between CP and cPGM in vitro (Figure 4B). Taken together, the Y2H, BiFC and GST pull‐down assays confirmed the interaction between PVY CP and cPGM.

### 2.3 Subcellular Localization of PVY‐CP and cPGM and Their Co‐Localization In Planta

Potyviral CPs have been shown to localize predominately in the cytosol, forming aggregates that facilitate the virus assembly and cell‐to‐cell movement (Mäkinen and Hafren 2014; Vozelj et al. 2025). To better understand the role of CP and cPGM in the host–virus interactions, subcellular localization assays were carried out. CP‐GFP fluorescence was observed predominately in the cytosol, as aggregates, but not in the nuclei (GFP fluorescence not merging with signal from DAPI), whereas fluorescence of the free GFP (control) was observed throughout the whole cell including cytosol and nuclei (Figure 5). These results demonstrate that, similar to other potyviral proteins, PVY^NTN^ CP localizes in the cytosol and forms aggregates. cPGM‐YFP fluorescence was observed predominantly in the cytosol as well as in nuclei (Figure 5).

**FIGURE 5:**
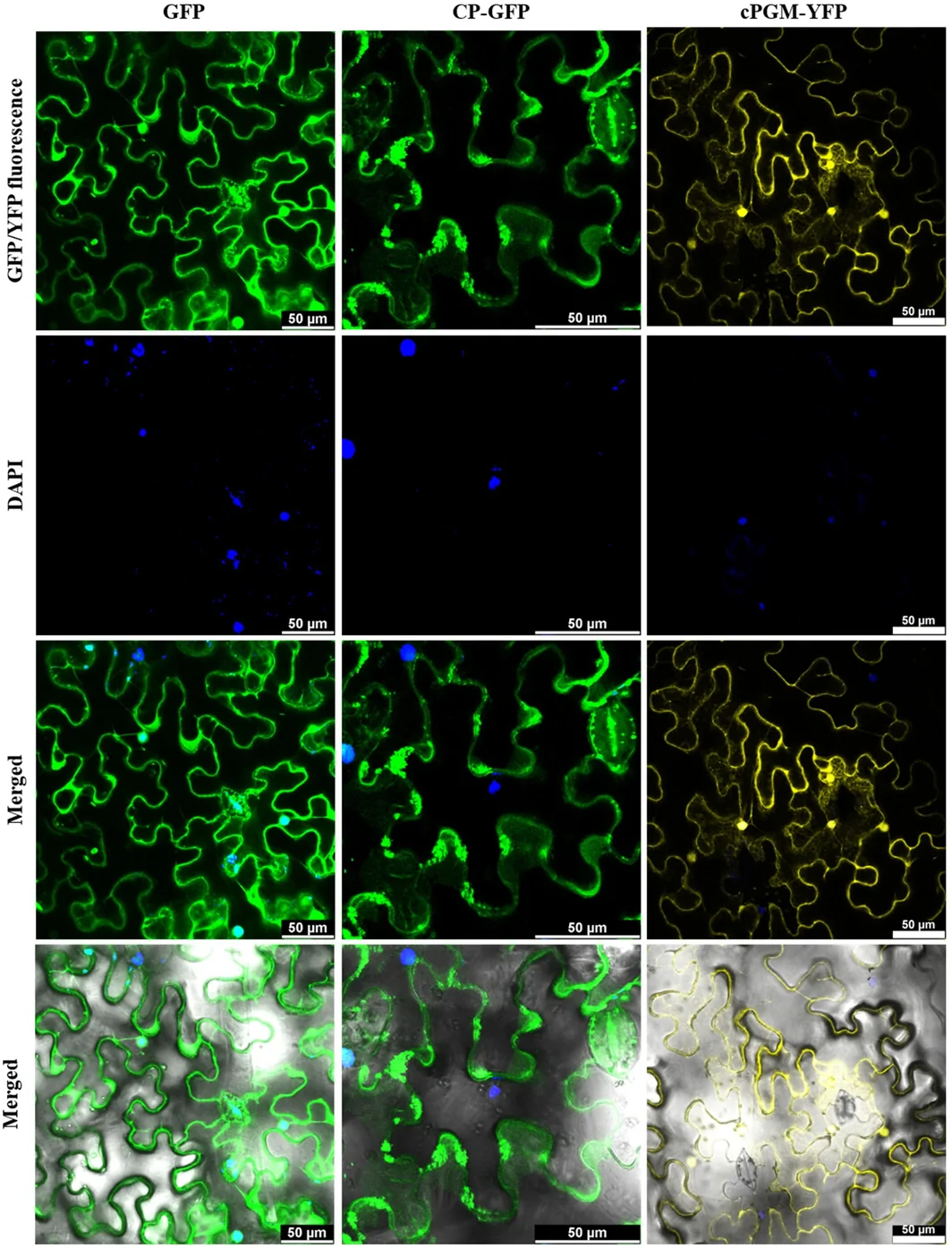
Subcellular localization of potato virus Y‐encoded coat protein (CP). *Agrobacterium tumefaciens* cells harbouring the pCAMBIA1302 vector or the pCAMBIA1302‐CP expression constructs were infiltrated into the abaxial surface of the leaves of 3‐week‐old *Nicotiana benthamiana* plants. The nuclei of the epidermal cells were stained with 4′,6‐diamidino‐2‐phenylindole (DAPI). The expression of free green fluorescent protein (GFP), CP‐GFP, and cytosolic phosphoglucomutase (cPGM)‐YFP recombinant proteins was observed in the leaves infiltrated with pCAMBIA1302 vector, pCAMBIA1302‐CP, and pEARLEY101Gate‐cPGM expression constructs, respectively. Images in rows 1, 2, 3 and 4 showing the GFP/YFP fluorescence image, DAPI fluorescence image, merged GFP and DAPI fluorescence image, and merged brightfield with GFP/YFP and DAPI fluorescence image, respectively. Scale bar represents 50 μm.

To investigate whether CP affects the subcellular distribution of cPGM, co‐localization analysis was performed in *N. benthamiana* leaves expressing cPGM‐YFP alone or co‐expressing cPGM‐YFP and CP‐GFP. When expressed alone, cPGM‐YFP fluorescence was observed predominantly in the cytosol as well as in nuclei. However, in the presence of CP, cPGM fluorescence accumulated in cytosolic aggregates associated with CP, resembling the structures observed in the BiFC assay (Figure 6). These observations suggest that CP expression alters the subcellular distribution of cPGM, potentially promoting its recruitment to CP‐associated aggregates.

**FIGURE 6:**
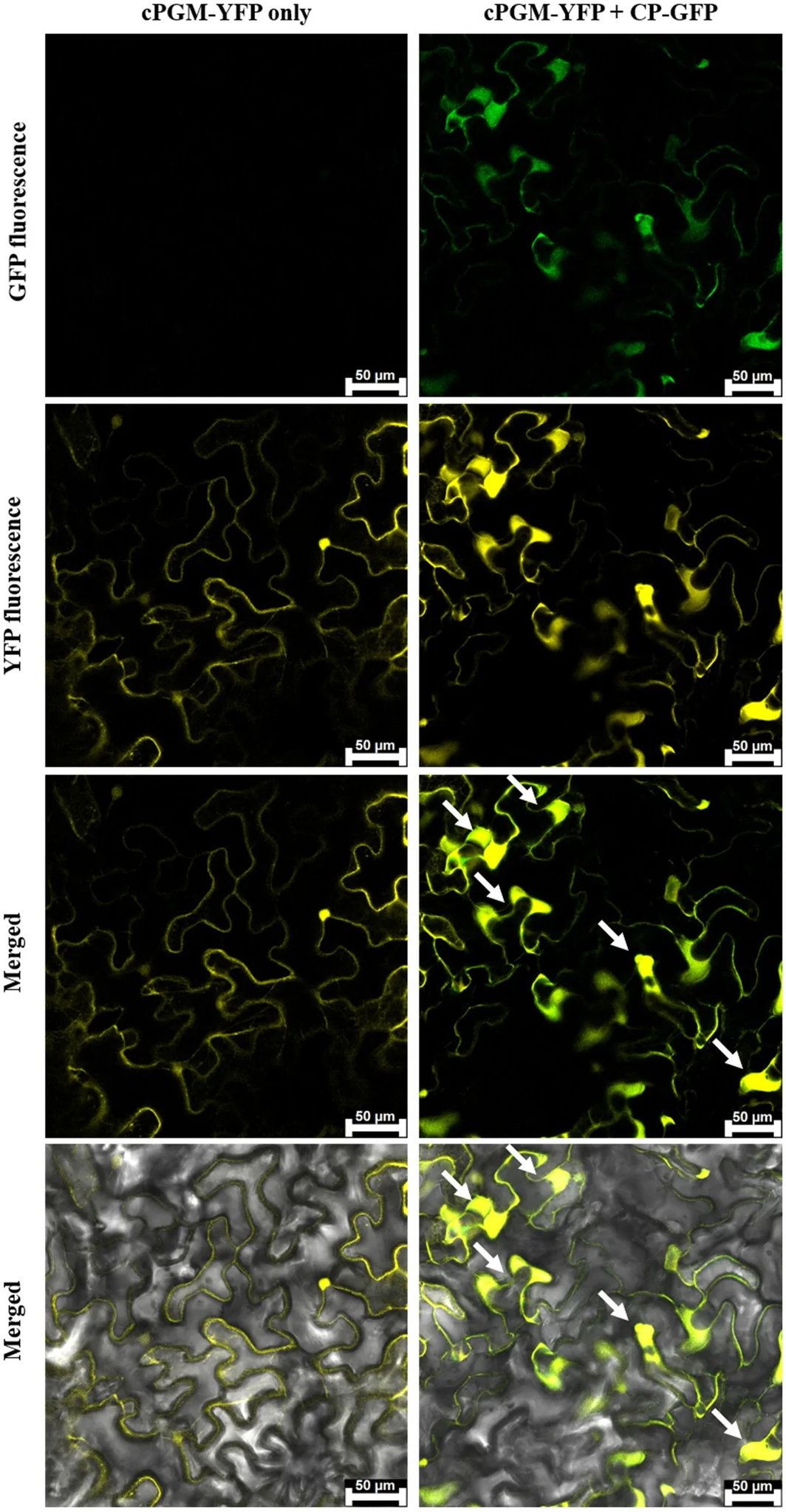
Potato virus Y‐encoded coat protein (CP) alters the subcellular distribution of cytosolic phosphoglucomutase (cPGM) in *Nicotiana benthamiana*. Co‐localization analysis was performed by infiltrating the leaves of 3‐week‐old *N. benthamiana* plants with *Agrobacterium tumefaciens* cells containing constructs expressing cPGM‐YFP alone or both cPGM‐YFP and CP‐GFP. The epidermal cells of the infiltrated leaves were visualized under a confocal microscope between 48 and 72 h post‐infiltration. Images in rows 1, 2, 3 and 4 show the GFP fluorescence image, YFP fluorescence image, merged GFP and YFP fluorescence image, and merged brightfield with GFP and YFP fluorescence image, respectively. Arrows indicate CP‐associated cytoplasmic aggregates. Scale bar represents 50 μm.

### 2.4 Transient Silencing of Endogenous *cPGM* Reduces PVY Accumulation

The endogenous *cPGM* in *N. benthamiana* plants was transiently silenced by agroinfiltration using tobacco rattle virus (TRV)‐based silencing constructs. The *cPGM*‐silenced plants did not exhibit any discernible phenotypic differences compared to pTRV‐infiltrated control plants (Obrępalska‐Stęplowska et al. 2018). Silencing of the *cPGM* gene was confirmed by marked reduction in *cPGM* mRNA level in pTRV‐*cPGM* infiltrated plants compared to pTRV‐infiltrated plants (Figure S4). To investigate the biological significance of CP–cPGM interaction, *cPGM*‐silenced and control plants were inoculated with PVY at 10 dpi of TRV‐based silencing constructs. At 15 dpi, PVY‐inoculated pTRV‐control plants showed typical symptoms including downward leaf curling and stunted growth, whereas PVY‐inoculated *cPGM*‐silenced plants showed downward leaf curling symptoms without any stunting (Figure 7A,B). PVY symptoms appeared in both pTRV control and *cPGM*‐silenced plants around 8–9 dpi; however, PVY‐infected *cPGM*‐silenced plants exhibited mild symptom severity compared to PVY‐infected pTRV control plants (Figure 7A,B). At 15 dpi, the relative levels of PVY were assessed in the upper, non‐inoculated leaves of the PVY‐infected *cPGM*‐silenced and pTRV‐control *N. benthamiana* plants by double antibody sandwich (DAS)‐ELISA. PVY accumulation level in the upper leaves of the PVY‐infected *cPGM*‐silenced plants was found to be significantly reduced compared to that of PVY‐infected pTRV‐control plants (Figure 7C). PVY viral RNA levels in the upper, non‐inoculated leaves of the PVY‐infected *cPGM*‐silenced and pTRV‐control *N. benthamiana* plants were determined by reverse transcription‐quantitative PCR (RT‐qPCR). Consistently, at 15 dpi, the PVY viral RNA levels were also found to be decreased in the PVY‐infected *cPGM*‐silenced plants compared to that of in the PVY‐infected pTRV‐control plants (Figure 7D). These results indicated that silencing of *cPGM* impairs the accumulation of PVY in *N. benthamiana* plants.

**FIGURE 7:**
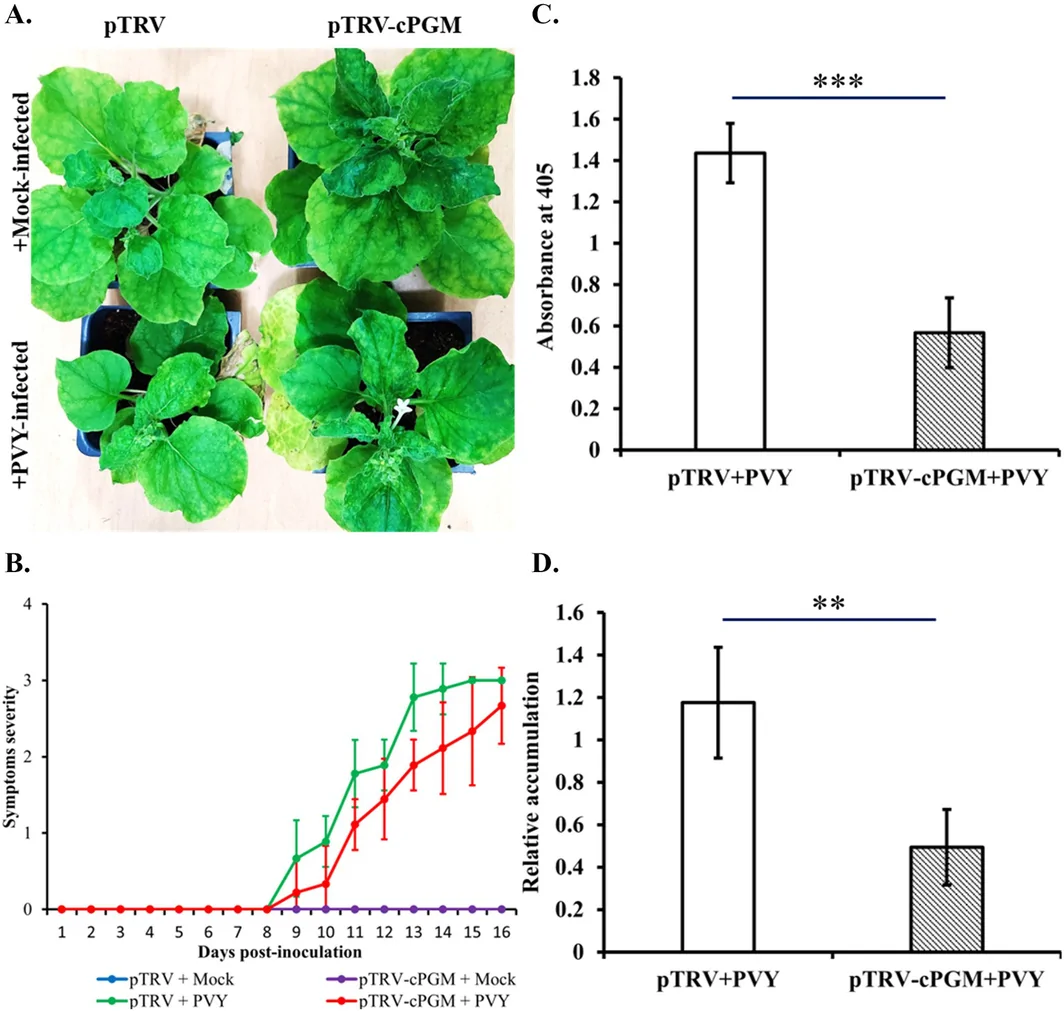
Silencing of *cytosolic phosphoglucomutase* (*cPGM*) interferes with potato virus Y (PVY) accumulation. (A) PVY induced symptoms on *cPGM*‐silenced or pTRV‐infiltrated *Nicotiana benthamiana* plants at 15 days post‐inoculation (dpi). (B) Appearance of PVY‐induced symptoms at different times after inoculation on *cPGM*‐silenced or pTRV‐infiltrated *N. benthamiana* plants. Infected *N. benthamiana* plants were visually scored for PVY symptoms on a scale of 0–4 (0 = no symptoms, 1 = mild leaf curling and/or mild mosaic, 2 = severe leaf curling and/or typical chlorosis, 3 = mild necrosis and/or stunted growth, 4 = severe necrosis). Error bar represents standard deviation. Relative PVY viral CP level (C) and viral RNA levels (D) in *cPGM*‐silenced or pTRV‐infiltrated control plants, determined by double antibody sandwich‐ELISA and reverse transcription‐quantitative PCR, respectively. **p < 0.01, ***p < 0.001 as determined by one‐way ANOVA.

### 2.5 Transient Expression of PVY *CP* Alters Levels of Key Carbohydrate Metabolites

To investigate whether PVY CP alone contributes to host metabolic reprogramming, we transiently expressed *CP‐GFP* in *N. benthamiana* leaves by agroinfiltration with the pCAMBIA1302‐*CP* expression construct. Leaves infiltrated with the empty pCAMBIA1302 vector served as controls. At 48 h post‐infiltration, leaf tissues were harvested for GC–MS‐based metabolomic analysis. The relative abundance of the key carbohydrate metabolites, glucose‐6‐phosphate and fructose‐6‐phosphate was determined. Although the fold changes in these metabolites were less pronounced than those observed during PVY infection, both glucose‐6‐phosphate and fructose‐6‐phosphate were consistently increased in *CP*‐expressing leaves compared with vector controls (Figure S5). These results suggest that expression of PVY CP alone is sufficient to induce metabolic changes similar to those observed during PVY infection, supporting a direct role for CP in host metabolic reprogramming.

## 3 Discussion

Host metabolic reprogramming has been well documented in animal pathogen systems. These metabolic changes are not merely consequences of virus infection but are recognized as virus‐driven processes that support its replication. For instance, several human‐infecting DNA and RNA viruses stimulate glycolytic, TCA cycle, glutamine utilization and nucleotide biosynthesis to fulfil the high energy and biosynthetic demands of virion production (Goodwin et al. 2015; Thaker et al. 2019). Notably, these virus‐driven metabolic changes are mediated by targeting key regulatory mechanisms. On the other hand, the intercellular bacterial pathogen *Mycobacterium tuberculosis* (MTB) reprogrammes host macrophage primary metabolism by suppressing the glycolytic flux to support its persistence and replication (Ehrt and Rhee 2013). MTB suppresses host glycolysis by targeting and repressing phosphofructokinase‐M, a rate‐limiting enzyme in the glycolytic pathway (Hackett et al. 2020). Several studies on animal virus‐driven metabolic reprogramming provide a conceptual framework for interpreting the metabolic changes observed during plant virus infection (Thaker et al. 2019; Goodwin et al. 2015; Munger et al. 2008; Yogev et al. 2014; Fontaine et al. 2015).

In the plant cell, the relative levels of glucose‐1‐phosphate and glucose‐6‐phosphate provide clues about the direction of carbohydrate metabolism (Stitt and Zeeman 2012). Glucose‐1‐phosphate is utilized in different biosynthetic pathways, including cellulose, hemicellulose, pectin and sucrose synthesis in the cytosol, as well as starch synthesis in chloroplasts. On the other hand, the increased level of glucose‐6‐phosphate directs the glucose metabolism towards respiratory pathways for energy production and the pentose phosphate pathway for the generation of biosynthetic precursors and NADPH required for nucleic acid synthesis. In the cytosol, carbohydrate partitioning is regulated by cPGM, an enzyme that catalyses the reversible interconversion of glucose‐1‐phosphate to glucose‐6‐phosphate (Fettke et al. 2010; Stitt and Zeeman 2012).

Carbon fixation in the Calvin cycle as well as starch mobilization majorly contribute to increased glucose‐6‐phosphate, a central molecule of carbohydrate that acts as a signal and substrate for the glycolytic pathway. Elevated levels of glucose‐6‐phosphate and its immediate downstream metabolite fructose‐6‐phosphate in leaves of the PVY‐infected plants indicate that carbohydrate metabolism is directed towards respiratory pathway. Glucose‐6‐phosphate is a key metabolic branch point that enters glycolysis for ATP production or the pentose phosphate pathway for NADPH and ribose‐5‐phosphate synthesis. Therefore, the accumulation of glucose‐6‐phosphate observed during PVY infection is consistent with an enhanced metabolic capacity to support the energetic and biosynthetic demands associated with viral genome replication. PVY infection is also associated with remarkable alterations in amino acid metabolism, with either elevated levels of asparagine, glutamic acid, glutamine, glycine, lysine, proline, serine and tyrosine and xylose or reduced levels of aspartic acid, isoleucine, methionine, threonic acid and valine. These alterations are consistent with increased carbon flux towards glycolysis and TCA cycles, which supply precursors for amino acid biosynthetic pathways including glycine, aspartate and glutamine.

Aspartate and glutamine are key amino acids that provide essential nitrogen and carbon atoms for the synthesis of purine and pyrimidine bases. Serine serves as a major source of one‐carbon units by donating a methylene group to tetrahydrofolate during its conversion to glycine, a reaction catalysed by serine hydroxy methyltransferase (Appling 1991; Hanson and Roje 2001). In the present study, the elevated levels of glycine accompanied by reduced serine abundance suggest a potential increase in tetrahydrofolate‐dependent one‐carbon metabolism. Glycine, both directly and through tetrahydrofolate‐mediated one‐carbon metabolism, contributes carbon units at multiple steps of nucleotide biosynthesis. The increased levels of glycine and glutamine detected during PVY infection may therefore reflect an expanded pool of nucleotide precursors, consistent with an increased demand for nucleotide synthesis associated with viral genome replication.

Similar to animal viruses, plant viruses are reported to manipulate host respiratory metabolism to support their replication. Similar to previous reports on BMV, CMV, SCMV, TMV, TRV and ZYMV, our findings indicate that PVY infection is associated with reprogramming of host carbohydrate and amino acid metabolism (Shalitin and Wolf 2000; Jiang et al. 2022; López‐Gresa et al. 2012; Xu et al. 2008; Blua et al. 1994). The present study provides first mechanistic evidence that a plant virus‐encoded protein can manipulate host primary metabolism by targeting a key regulatory enzyme controlling carbohydrate partitioning.

We identified that PVY CP protein interacts with cPGM protein, an enzyme that regulates carbohydrate metabolism in the cytosol by controlling the interconversion of glucose‐6‐phosphate and glucose‐1‐phosphate, thereby partitioning the carbon flux between respiratory metabolism, the pentose phosphate pathway and several biosynthetic pathways. The interaction between PVY CP and cPGM provides a mechanistic link between viral infection and host metabolic reprogramming. Because glucose‐6‐phosphate serves as a substrate for both glycolysis and the pentose phosphate pathway, modulation of cPGM activity could alter cytosolic sugar‐phosphate homeostasis and increase the production of ATP, NADPH and ribose‐5‐phosphate required for viral RNA synthesis.

BiFC assay showed that CP and cPGM proteins interact predominately in the cytosol. Consistent with this observation, subcellular localization analyses showed that PVY CP protein localizes to the cytosol, further supporting the physiological relevance of the CP–cPGM interaction in modulating cytosolic carbohydrate metabolism during PVY infection. The mechanism by which CP–cPGM interaction fine tunes cPGM activity to redirect the carbohydrate flux towards respiratory pathways is not yet understood. CP forms cytoplasmic aggregates and interacts with cPGM, suggesting that viral infection possibly recruits cPGM to viral replication sites and influences its substrate accessibility, thereby modulating local metabolic flux. Our colocalization analysis revealed that CP alters the subcellular distribution of cPGM, which accumulates in CP‐associated cytosolic aggregates in the presence of CP. This CP‐induced redistribution of cPGM may influence its availability and function in host carbohydrate metabolism, although the effects on cPGM enzymatic activity and metabolic flux remain to be determined.

Interestingly, findings from this study showed reduced disease severity and PVY accumulation in *cPGM* gene‐silenced *N. benthamiana*. Changes in cPGM protein levels were positively correlated with PVY disease severity and virus accumulation, indicating that elevated levels of cPGM may be recruited and used by PVY through CP–cPGM interaction. Expression of PVY CP alone resulted in increased glucose‐6‐phosphate and fructose‐6‐phosphate levels, but not completely similar during PVY infection. These findings suggest that CP is a major viral determinant contributing to metabolic reprogramming, whereas the more pronounced metabolic changes observed during PVY infection likely result from the combined effects of multiple viral proteins and host responses.

Based on these findings, we propose a working model of PVY CP–cPGM interaction. PVY CP interacts with cPGM in the cytosol and alters its subcellular distribution, potentially affecting its role in the cytosolic sugar‐phosphate partitioning towards glucose‐6‐phosphate and fructose‐6‐phosphate, thereby promoting flux through respiratory, the pentose phosphate pathways and altering amino acid metabolism. Altered amino acid metabolism, with elevated glycine and glutamine levels, contributes to synthesis of building blocks required for nucleotide synthesis. Together, our metabolomic, protein interaction, functional genomics and CP expression analyses support a model in which PVY CP targets cPGM to reprogramme host primary metabolism, thereby promoting metabolically conditions favourable for nucleotide biosynthesis, viral replication and disease development (Figure 8). While this model provides a conceptual framework for understanding PVY‐mediated metabolic manipulation, the precise molecular mechanisms by which CP–cPGM interaction influences enzyme activity, metabolite flux, or regulatory signalling remain to be elucidated.

**FIGURE 8:**
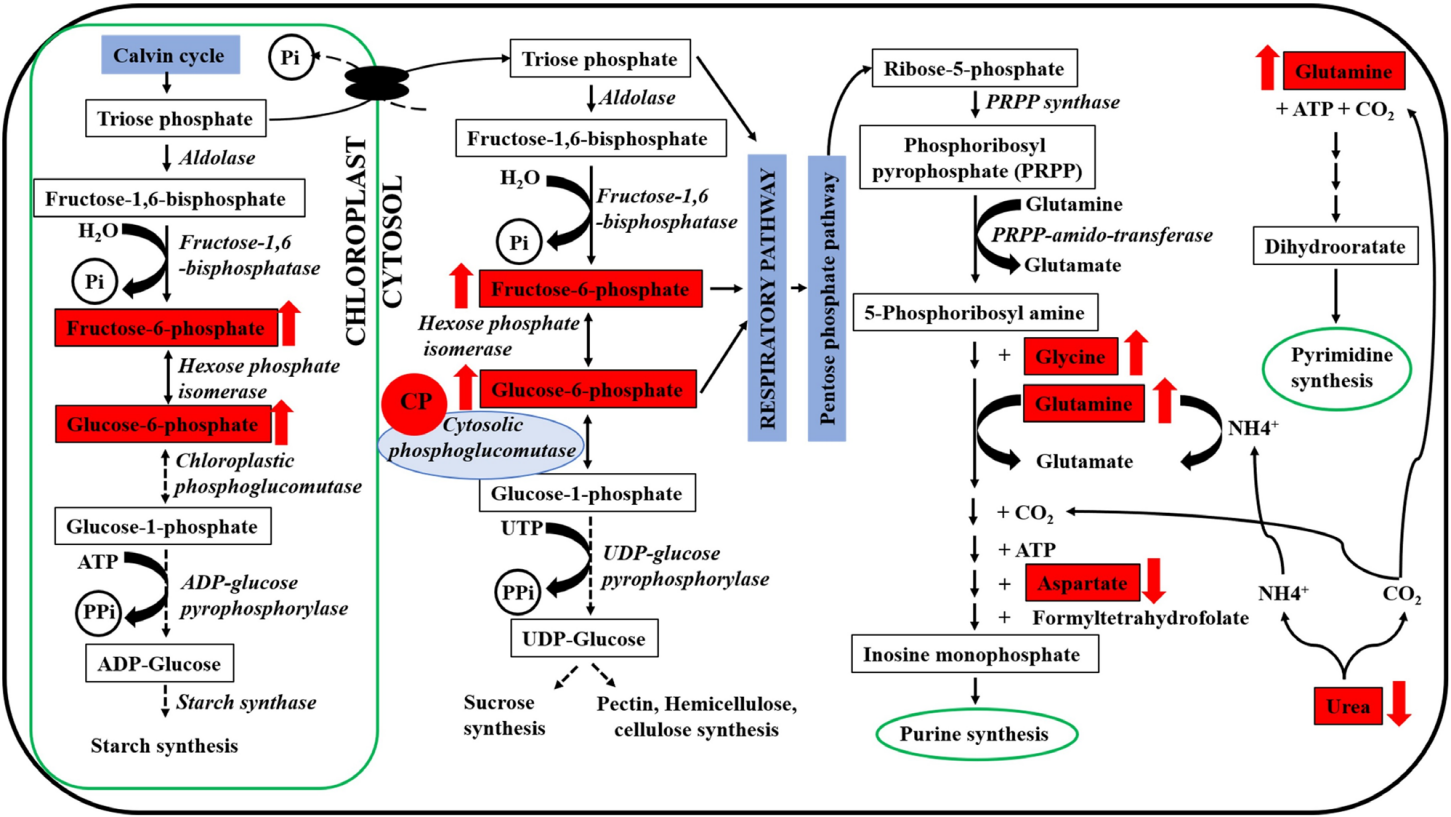
Proposed working model of potato virus Y (PVY) coat protein (CP)‐cytosolic phosphoglucomutase (cPGM) interaction. cPGM is an enzyme that controls carbohydrate partitioning by catalysing the interconversion of glucose‐1‐phosphate to glucose‐6‐phosphate. Glucose‐1‐phosphate is primarily involved in biosynthetic pathways, while glucose‐6‐phosphate is primarily involved in respiratory pathways. PVY CP interacts with cPGM in the cytosol. This interaction possibly alters cytosolic sugar‐phosphate partitioning, favouring the accumulation of glucose‐6‐phosphate and fructose‐6‐phosphate, thereby promoting metabolic flux through respiratory pathways and the pentose phosphate pathway. Enhanced flux through these pathways could increase the availability of energy, reducing equivalents and biosynthetic precursors. Concomitant changes in amino acid metabolism, including elevated levels of glycine and glutamine, may reflect altered nucleotide precursor availability associated with viral genome replication. Upward and downward arrows indicate the elevated or reduced metabolite levels, respectively, during infection by PVY. Solid and dashed lines indicate proposed metabolic fluxes and regulation, respectively, during PVY infection.

## 4 Experimental Procedures

### 4.1 Plant Inoculations

The symptomatic leaves of the *N. benthamiana* plants that were previously infected with PVY^NTN^ and confirmed by ELISA were used as the source of PVY inoculum. *N. benthamiana* plants were infected with PVY following the standard mechanical inoculation protocol as previously described by Gnanasekaran et al. (2023). Tissue sap was prepared by homogenizing 1 g of PVY‐infected leaves in 10 mL of 100 mM sodium phosphate buffer (pH 7.2) containing 0.4% (v/v) β‐mercaptoethanol and carborundum at a 1:10 ratio (w/v) was added as abrasive. Sap extracted from PVY‐infected leaves was used for PVY inoculation, while the sap from leaves of healthy *N. benthamiana* plants was used for mock inoculation. Mechanical inoculation was carried out by gently rubbing the sap inoculum on the adaxial side of leaves of the *N. benthamiana* plants. PVY disease symptom severity was evaluated visually and scored on scale ranging from 0 to 5 (0 = no visible symptoms, 1 = mild leaf curling and/or mild mosaic, 2 = severe leaf curling and/or typical chlorosis, 3 = mild necrosis and/or stunted growth, 4 = severe necrosis, 5 = leaf and/or shoot tip death).

### 4.2 GC–MS Analysis

GC–MS analysis was performed using the leaves of the mock‐ or PVY‐infected *N. benthamiana* plants collected at 14 dpi.

Leaf metabolite extraction was carried out following the protocol previously described by Lee and Fiehn (2008). From each sample, 100 mg of frozen leaf tissue powder was suspended in 1 mL of extraction solvent containing methanol, isopropanol and water in a 5:2:2 ratio. Samples were vortexed for 10 min and sonicated for 10 min. The cell debris was pelleted by centrifugation at 21,000 *g* for 10 min, and 200 μL of supernatant from each sample was vacuum dried. The dried extracts were reconstituted in 200 μL of 50% aqueous acetonitrile, vortexed, sonicated, centrifuged and vacuum dried again prior to derivatization.

Each dried extract was dissolved in 10 μL of *O*‐methoxylamine hydrochloride (Sigma‐Aldrich) by vortexing for 5 min and incubated at 30°C with 1000 rpm shaking for 90 min. Following that, silylation was carried using 90 μL of *N*‐methyl‐*N*‐(trimethylsilyl)‐trifluoroacetamide (MSTFA) containing 1% trimethylchlorosilane (TMCS; Thermo‐Pierce). Samples were incubated at 37°C with 1000 rpm shaking for 30 min. The derivatized samples were then transferred into 250 μL glass inserts (Agilent Technologies) for GC–MS analysis.

GC–MS analysis was performed using a Pegasus 4D time‐of‐flight mass spectrometer (LECO) equipped with a Gerstel MPS2 autosampler and an Agilent 7890A gas chromatograph. Mass spectra were recorded using electron impact ionization at 70 eV with a scan rate of 17 spectra per second. Chromatographic peak alignment and spectral comparisons were performed using the Statistical Compare function of ChromaTOF software (LECO).

Metabolites were identified by spectral matching against the Fiehn Library (Kind et al. 2009) with a similarity threshold of 600; tentative identifications above 700 were accepted. Matches were manually validated using an in‐house standard library (Institute of Biological Chemistry, Washington State University). Metabolite abundances were normalized to both internal control: D6‐salicylic acid and to the sample fresh weight. The peak intensities of different metabolites obtained from GC–MS (Table S1) were analysed using the MetaboAnalyst v. 5.0.

### 4.3 Cloning and Generation of Recombinant Plasmid Constructs

To generate the entry clone, pDONR201‐CP, the coding sequence of CP of PVY necrotic strain PVY^NTN^ (GenBank accession no. ON866943) (Gnanasekaran et al. 2023) was PCR‐amplified using a primer pair B1NTNCP FP/RP, and cloned into the entry vector pDONR201. To generate the pDONR201‐cPGM construct, the full‐length coding sequence of cPGM (GenBank accession no. PZ273815) was PCR‐amplified from the cDNA of *N. benthamiana* leaves by using the specific primer pair PGMcy FP/RP and cloned into the pDONR201 vector. Similarly, the pDONR201‐pPGM construct was generated by PCR amplification of the full‐length coding sequence of chloroplastic phosphoglucomutase (pPGM) (GenBank accession no. PZ273816) using the specific primer pair PGMch FP/RP and cloning into the pDONR201 vector.

Y2H and BiFC assays constructs were generated by in vitro recombination‐mediated mobilization from an entry clone to the destination vector by using Gateway LR Clonase enzyme mix (Thermo Fisher Scientific) by following the manufacturer's instructions. The pGBKT7‐CP constructs were generated by mobilizing the CP from pDONR201‐CP to the pGBKT7‐GW vector. The pGADT7‐cPGM and pGADT7‐pPGM constructs were generated by mobilizing the cPGM and pPGM, respectively, from pDONR201‐cPGM and pDONR201‐pPGM to the pGADT7‐GW vector. BiFC construct nYFP‐CP was generated by mobilizing the CP from pDONR201‐CP to the pSITE‐BiFC‐nEYFP‐C1 vector (Tripathi et al. 2015; Chakrabarty et al. 2007; Goodin et al. 2007). Similarly, cYFP‐cPGM and cYFP‐pPGM constructs were generated by mobilizing the cPGM and pPGM from pDONR201‐cPGM and pDONR201‐pPGM, respectively, to the pSITE‐BiFC‐cEYFP‐C1 vector (Tripathi et al. 2015; Chakrabarty et al. 2007; Goodin et al. 2007).

To generate CP‐GFP localization construct (pCAMBIA1302‐CP), the full‐length coding sequence of CP was amplified with the primer pair CPLo FP/RP, cloned into pJET1.2 and subcloned in NcoI and BglII restriction sites of the pCAMBIA1302 vector. The cPGM‐YFP localization construct (pEARLEY101Gate‐cPGM) was generated by mobilizing the coding sequence of cPGM from pDONR201‐cPGM into the pEarleyGate101 vector (ABRC). To generate GST‐CP protein expression construct (pGEX‐6p2‐CP), the full‐length coding sequence of CP was amplified with the primer pair CPEx FP/RP, cloned into pJET1.2 and subcloned in EcoRI and SalI restriction sites of the pGEX‐6p2 vector. To generate MBP‐cPGM protein expression construct (pMAL‐c2X‐cPGM), the full‐length coding sequence of cPGM was amplified with the primer pair EXPGMcyFP/pEXPGMcyRP, cloned into pJET1.2 and subcloned in BamHI and SalI restriction sites of the pMAL‐c2X vector. To generate the pTRV‐cPGM‐silencing construct, cPGM‐fragment (304 bp) was amplified using the primer pair SiPGMcy FP/RP and cloned in antisense orientation in the pTRV2‐GW vector. To generate the pGR106‐cPGM expression construct, the full‐length coding sequence of cPGM was amplified with the primer pair PExPGMcy FP/R, cloned into pJET1.2 and subcloned in ClaI and SalI restriction sites of the pGR106 vector. Sequences of all the primers used in this study are listed in Table S2.

### 4.4 Y2H Assay

The pGBKT7‐CP construct having the coding sequence of coat protein (CP) of PVY^NTN^ in‐frame with the yeast GAL4 binding domain protein was used as a bait construct for the Y2H library screening (Gnanasekaran et al. 2023). The pGBKT7‐CP construct was transformed into *S. cerevisiae* Y2HGold cells and used as a bait for screening the *N. benthamiana* Y2H cDNA library. Y2H library screening was carried out as described in our previous study (Gnanasekaran et al. 2023; Gnanasekaran and Pappu 2023c).

One‐to‐one Y2H assays were performed by co‐transforming pGBKT7‐CP and pGADT7‐cPGM plasmids into *S. cerevisiae* Y2HGold cells. A pGBKT7‐P53 plus pGADT7‐TAg plasmid combination was used as a positive control, while a pGBKT7‐CP plus pGADT7 vector, pGBKT7 vector plus pGADT7‐cPGM, and pGBKT7 vector plus pGADT7 vector combination was used as the negative control. The interaction was tested by growing the yeast co‐transformants on synthetic screening medium lacking Leu, Trp and His and containing 1 mM 3‐AT and X‐α‐Gal at 30°C for 4–5 days. Similarly, one‐to‐one Y2H assays were performed to test the interaction between CP and pPGM.

### 4.5 BIFC Assay

BIFC assays were performed as described previously (Tripathi et al. 2015; Gnanasekaran et al. 2019; Gnanasekaran and Pappu 2023a) using pSITE vectors containing the N‐ and C‐terminal halves of enhanced yellow fluorescent protein (EYFP), nYFP and cYFP. Empty BIFC vectors harbouring nYFP and cYFP, and the BIFC constructs harbouring nYFP‐CP and cYFP‐cPGM were individually transformed into *A. tumefaciens* GV3101. Leaves of 3‐week‐old *N. benthamiana* plants were co‐infiltrated with *Agrobacterium* cells containing nYFP‐CP and cYFP‐cPGM. Similarly, *Agrobacterium* cells containing nYFP and cYFP, nYFP‐CP and cYFP, and nYFP and cYFP‐cPGM negative combinations were infiltrated into leaves of *N. benthamiana* plants. At 48 h post‐infiltration, the abaxial epidermal cells of the infiltrated *N. benthamiana* leaves were examined using a Leica TCS SP8 X confocal laser‐scanning microscope with a 20× objective lens, and the LAS X software (Leica Microsystems). For detection of chlorophyll autofluorescence, 543 nm excitation and a 650 nm emission filter were used. For detecting reconstituted yellow fluorescent protein (YFP), 514 nm excitation was used, and the emitted light was collected between 530 and 570 nm.

### 4.6 GST Pull‐Down Assay

The GST pull‐down assay was performed as described previously (Gnanasekaran and Pappu 2023b; Gnanasekaran et al. 2019), with modifications. GST and GST‐CP fusion proteins were expressed in *Escherichia coli* CodonPlus (DE3) using the pGEX‐6p2 vector and pGEX‐6p2‐CP expression constructs, respectively. MBP and MBP‐cPGM fusion proteins were expressed in *E. coli* CodonPlus (DE3) using the pMAL‐c2X vector and pMAL‐c2X‐cPGM expression constructs, respectively. Protein expression was induced with 0.1 mM isopropyl β‐D‐1‐thiogalactopyranoside for 3 h at 25°C. GST and GST‐CP proteins were purified using glutathione resin (G‐Biosciences), whereas MBP and MBP‐cPGM proteins were purified using amylose resin (New England Biolabs), following the manufacturers' instructions. Equal amounts of purified proteins were mixed in the following combinations: GST with MBP, GST‐CP with MBP, GST with MBP‐cPGM and GST‐CP with MBP‐cPGM. The mixtures were incubated with pre‐equilibrated glutathione resin for 4 h at 4°C. The resin was then washed five times with wash buffer, and bound proteins were eluted. The 10% of the input proteins and the eluted proteins were analysed by SDS‐PAGE and western blotting using an anti‐MBP antibody (Sigma) to detect MBP and MBP‐cPGM proteins.

### 4.7 Subcellular Localization

Subcellular localization of PVY^NTN^ CP and cPGM proteins were carried out as described previously (Gnanasekaran et al. 2023; Gupta et al. 2022) using pCAMBIA1302‐CP and pEARLEY101Gate‐cPGM constructs. The plasmids of the subcellular localization constructs pCAMBIA1302 empty vector, pCAMBIA1302‐CP and pEARLEY101Gate‐cPGM constructs were individually transformed into *A. tumefaciens* GV3101 cells. The empty pEarleyGate101 vector does not express free YFP and was therefore not included control in the subcellular localization assay. The leaves of the 3‐week‐old *N. benthamiana* plants were agroinfiltrated with 0OD_600_ = 0.5 of *Agrobacterium* cells containing localization plasmids. At 48 h post‐infiltration, the abaxial epidermal cells of the infiltrated *N. benthamiana* leaves were examined using a Leica TCS SP8 X confocal laser‐scanning microscope using 20× objective lens, and the LAS X software (Leica Microsystems). To visualize the nuclei, the epidermal cells of the infiltrated leaves were stained with 4′,6‐diamidino‐2‐phenylindole (DAPI). For detection of DAPI fluorescence, 405 nm blue diode was used for excitation, and the emitted light was collected between 435 and 485 nm. For detection of GFP fluorescence, 488 nm excitation was used, and the emitted light was collected at 530 ± 15 nm. For detection of YFP fluorescence, 514 nm excitation was used and the emitted light was collected at 524–550 nm.

### 4.8 Co‐Localization of cPGM With CP

To study the subcellular distribution of cPGM in the presence of CP, leaves of 3‐week‐old *N. benthamiana* plants were infiltrated with *A. tumefaciens* GV3101 carrying the pEarleyGate101‐cPGM construct, either alone or in combination with the pCAMBIA1302‐CP construct. Leaves expressing cPGM‐YFP alone served as the control. cPGM‐YFP and CP‐GFP fluorescence was examined by confocal microscopy to assess the subcellular distribution and colocalization of cPGM and CP. Because GFP and YFP have overlapping emission spectra, a lambda scan was performed to identify suitable emission windows for distinguishing the two fluorescent signals. GFP fluorescence was detected using excitation at 483 nm and emission at 493–524 nm, whereas YFP fluorescence was detected using excitation at 514 nm and emission at 524–550 nm.

### 4.9 Silencing of Endogenous *cPGM* in *N. benthamiana* Plants

Transient silencing of endogenous *cPGM* was carried out as described previously (Gnanasekaran et al. 2023, 2019) using TRV‐based silencing vectors, pTRV1 (CD3‐1039) and pTRV2‐GW (CD3‐1041) vectors obtained from the Arabidopsis Biological Resource Center, OH, United States. The plasmids of the empty vector, pTRV1 and pTRV2, as well as the pTRV2‐*cPGM* silencing construct were transformed into *A. tumefaciens* EHA105. Silencing of endogenous *cPGM* in the 3‐week‐old *N. benthamiana* plants was achieved by co‐infiltration with *Agrobacterium* cells carrying pTRV1 and pTRV2‐*cPGM*. Plants with co‐infiltrated *Agrobacterium* carrying either pTRV1 and pTRV2‐*NbPDS*, or pTRV1 and pTRV2 combination served as positive and negative controls, respectively. The infiltrated plants were grown at 22°C, 70% relative humidity and in 16 h light/8 h dark with light intensity of 100 μmol/m^2^/s.

### 4.10 ELISA

The relative accumulation of viral load present in the systemically infected top leaves of the PVY‐infected plants was analysed by DAS‐ELISA using an ELISA reagent set (SRA 20001, Agdia). DAS‐ELISA samples were prepared by grinding 1 g of leaf tissue in 10 mL of general extract buffer (Agdia). For measuring the viral titre by DAS‐ELISA, 100 μL of leaf samples were used with two technical replicates and three biological replicates for each treatment.

### 4.11 Total RNA Extraction and RT‐qPCR

Total RNAs from the systemically infected top leaves of the PVY‐infected plants were extracted using Quick RNA Mini prep kit (Zymo Research) and treated with DNase I (Sigma). The cDNA synthesis was carried out using the DNase I treated total RNA and iScript cDNA synthesis kit (Bio‐Rad). The qPCR analysis was carried out as follows: as described previously (Gnanasekaran et al. 2023) and by using protein phosphatase 2A (*PP2A*) as an internal reference gene. RT‐qPCR analysis performed in this study was carried out with three biological and three technical replicates. Statistical significance was determined by one‐way ANOVA.

## Author Contributions

**Prabu Gnanasekaran:** conceptualization, investigation, methodology, validation, visualization, writing – review and editing, software, formal analysis, data curation, writing – original draft. **Ying Zhai:** methodology, data curation, formal analysis, validation, conceptualization. **Hanu R. Pappu:** conceptualization, investigation, funding acquisition, writing – review and editing, project administration, supervision, resources.

## Funding

This work is supported by Hatch Project #1016563 from the USDA's National Institute of Food and Agriculture.

## Conflicts of Interest

The authors declare no conflicts of interest.

## Supporting information


**Figure S1:** Levels of metabolites elevated in potato virus Y (PVY)‐infected plants. Bar plots showing the relative abundance of elevated metabolite in the PVY‐infected plants compared with the mock‐inoculated controls. Bar plots were generated using the MetaboAnalyst v. 5.0 and show the mean and standard deviation of metabolite levels from four biological replicates of mock‐ or PVY‐infected plants.

**Figure S2:** Levels of metabolites reduced in potato virus Y (PVY)‐infected plants. Bar plots showing the relative abundance of reduced metabolite in the PVY‐infected plants compared with the mock‐inoculated controls. Bar plots were generated using the MetaboAnalyst v. 5.0 and show the mean and standard deviation of metabolite levels from four biological replicates of mock‐ or PVY‐infected plants.

**Figure S3:** Potato virus Y‐encoded coat protein (CP) interacts with *Nicotiana benthamiana* cytosolic phosphoglucomutase (cPGM) in planta. *Agrobacterium tumefaciens* cells containing bimolecular fluorescence complementation assay constructs were co‐infiltrated into the abaxial surface of leaves of 3‐week‐old *N. benthamiana* plants. The epidermal cells of the infiltrated leaves were nuclear stained using 4′,6‐diamidino‐2‐phenylindole (DAPI) and visualized under a confocal microscope between 48 and 72 h post‐infiltration. The reconstituted yellow fluorescent protein (YFP) fluorescence was checked in the leaves co‐infiltrated with nYFP‐CP + cYFP‐cPGM and nYFP‐CP + cYFP‐pPGM. The leaves co‐infiltrated with either nYFP + cYFP, nYFP‐CP + cYFP, nYFP + cYFP‐cPGM, or nYFP + cYFP‐pPGM served as negative controls. Images in columns 1, 2, 3, 4 and 5 showing the bright field image, YFP fluorescence image, chlorophyll autofluorescence image, merged YFP and chlorophyll autofluorescence image, and merged brightfield with YFP and chlorophyll autofluorescence image, respectively. Scale bar represents 50 μm.

**Figure S4:** Comparative level of *cytosolic phosphoglucomutase* (*cPGM*) transcripts in tobacco rattle virus (pTRV)‐infiltrated and cPGM‐silenced *Nicotiana benthamiana*. Relative accumulation of transcripts level of *cPGM* in *cPGM*‐silenced or pTRV‐infiltrated control plants at 10 days post‐infiltration determined by quantitative real‐time PCR. ***p < 0.001 as determined by one‐way ANOVA.

**Figure S5:** Potato virus Y (PVY) coat protein (CP) induced metabolic changes in *Nicotiana benthamiana*. (A) Heatmap depicting the differentially altered metabolites in the leaves of the pCAMBIA1302‐CP and pCAMBIA1302 empty vector agroinfiltrated *N. benthamiana* plants at 48 h post‐infiltration (hpi). The heatmap was generated using MetaboAnalyst v. 6.0 and based on normalized metabolite intensities. Bar plots showing the relative abundance of key metabolite, glucose‐6‐phosphate (B) and fructose‐6‐phosphate (C) in the pCAMBIA1302‐CP infiltrated leaves compared with the pCAMBIA1302‐infiltrated controls. Bar plots were generated using the MetaboAnalyst v. 6.0 and show the mean and standard deviation of metabolite levels from three biological replicates.

**Table S1:** Gas chromatography–mass spectrometry (GC–MS) peak intensities of detected metabolites in mock‐ and potato virus Y‐infected leaves.

**Table S2:** List of primers used in this study.

## Data Availability

The datasets presented in this study can be found in online repositories/supporting information. Sequence generated in this study has been deposited in the GenBank database under accession numbers PZ273815 and PZ273816.
