# Supplementary material for: Potato Virus Y Coat Protein Interacts With Cytosolic Phosphoglucomutase and Reprogrammes Host Primary Metabolism During Disease Development
Source: Mol Plant Pathol. 2026 Nov 9;27(11):e70368. doi: 10.1111/mpp.70368 (PMC13748820; doi:10.1111/mpp.70368)
Supplement: Supplementary file 1 [file MPP-27-e70368-s002.docx]

**Figure S3. Potato virus Y-encoded coat protein (CP) interacts with *Nicotiana benthamiana* cytosolic phosphoglucomutase (cPGM) *in planta*.**

*Agrobacterium tumefaciens* cells containing bimolecular fluorescence complementation assay constructs were co-infiltrated into the abaxial surface of leaves of three-week-old *Nicotiana benthamiana* plants. The epidermal cells of the infiltrated leaves were nuclear stained using 4, 6-diamidino-2-phenylindole (DAPI) and visualized under a confocal microscope between 48 and 72 hours post-infiltration. The reconstituted yellow fluorescent protein (YFP) fluorescence was checked in the leaves co-infiltrated with nYFP-CP + cYFP-cPGM and nYFP-CP + cYFP-pPGM. The leaves co-infiltrated with either nYFP + cYFP, nYFP-CP + cYFP, nYFP + cYFP-cPGM, or nYFP + cYFP-pPGM served as negative controls. Images in columns 1, 2, 3, 4, and 5 showing the bright field image, YFP fluorescence image, chlorophyll autofluorescence image, merged YFP and chlorophyll autofluorescence image, and merged brightfield with YFP and chlorophyll autofluorescence image, respectively. Scale bar represents 50 µm.
