# Supplementary material for: Potato Virus Y Coat Protein Interacts With Cytosolic Phosphoglucomutase and Reprogrammes Host Primary Metabolism During Disease Development
Source: Mol Plant Pathol. 2026 Nov 9;27(11):e70368. doi: 10.1111/mpp.70368 (PMC13748820; doi:10.1111/mpp.70368)
Supplement: Supplementary file 3 [file MPP-27-e70368-s004.docx]

 **Figure S5. Potato virus Y (PVY) coat protein (CP) induced metabolic changes in *Nicotiana benthamiana*.** (A) Heatmap depicting the differentially altered metabolites in the leaves of the pCAMBIA1302-CP and pCAMBIA1302 empty vector agroinfiltrated *N. benthamiana* plants at 48 hours post-infiltration (hpi). The heatmap was generated using MetaboAnalyst version 6.0 and based on normalized metabolite intensities. Bar plots showing the relative abundance of key metabolite, glucose-6-phosphate (B) and fructose-6-phosphate (C) in the pCAMBIA1302-CP infiltrated leaves compared with the pCAMBIA1302-infiltrated controls. Bar plots were generated using the MetaboAnalyst version 6.0 and show the mean and standard deviation of metabolite levels from three biological replicates.
