# Supplementary material for: Potato Virus Y Coat Protein Interacts With Cytosolic Phosphoglucomutase and Reprogrammes Host Primary Metabolism During Disease Development
Source: Mol Plant Pathol. 2026 Nov 9;27(11):e70368. doi: 10.1111/mpp.70368 (PMC13748820; doi:10.1111/mpp.70368)
Supplement: Supplementary file 4 [file MPP-27-e70368-s005.docx]

**Figure S2. Levels of metabolites reduced in potato virus Y infected plants.** Bar plots showing the relative abundance of reduced metabolite in the potato virus Y (PVY)-infected plants compared with the mock-inoculated controls. Bar plots were generated using the MetaboAnalyst version 5.0 and show the mean and standard deviation of metabolite levels from four biological replicates of mock- or PVY-infected plants.
