# Supplementary material for: Potato Virus Y Coat Protein Interacts With Cytosolic Phosphoglucomutase and Reprogrammes Host Primary Metabolism During Disease Development
Source: Mol Plant Pathol. 2026 Nov 9;27(11):e70368. doi: 10.1111/mpp.70368 (PMC13748820; doi:10.1111/mpp.70368)
Supplement: Supplementary file 5 [file MPP-27-e70368-s006.docx]

***

**Figure S4. Comparative level of *cytosolic phosphoglucomutase* (*cPGM*) transcripts in tobacco rattle virus (pTRV)-infiltrated and cPGM-silenced *Nicotiana benthamiana*.** Relative accumulation of transcripts level of *cytosolic phosphoglucomutase (cPGM)* in cPGM-silenced or pTRV-infiltrated control plants at 10 days post infiltration determined by quantitative real-time PCR. *** indicates P-value < 0.001 as determined by one-way ANOVA.
