# Supplementary material for: Potato Virus Y Coat Protein Interacts With Cytosolic Phosphoglucomutase and Reprogrammes Host Primary Metabolism During Disease Development
Source: Mol Plant Pathol. 2026 Nov 9;27(11):e70368. doi: 10.1111/mpp.70368 (PMC13748820; doi:10.1111/mpp.70368)
Supplement: Supplementary file 6 [file MPP-27-e70368-s007.docx]

**Table S2**: List of primers used in this study.

| **Primer name** | **Sequence** |
| --- | --- |
| B1NTNCP FP | GGGGACAAGTTTGTACAAAAAAGCAGGCTCCATGGGAAATGACACAATCGATGCAGG |
| B1NTNCP RP | GGGGACCACTTTGTACAAGAAAGCTGGGTTCATGTTCTTGACTCCAAGTAGAG |
| PGMch FP | GGGGACAAGTTTGTACAAAAAAGCAGGCTCCATGGAGAATGCAATTACATTG |
| PGMch RP | GGGGACCACTTTGTACAAGAAAGCTGGGTTTGTGATGACGGTTGGCTTTTCTC |
| PGMcy FP | GGGGACAAGTTTGTACAAAAAAGCAGGCTCCATGGTGATGTTCAATATCACTCGC |
| PGMcy RP | GGGGACCACTTTGTACAAGAAAGCTGGGTTTGTAATAACAGTTGGGGCAGAAC |
| CPLo FP | CCATGGTAATGGGAAATGACACAATCG |
| CPLo RP | AGATCTACCATGTTCTTGACTCCAAGTAGAG |
| ExPGMcy FP | GGATCCATGGTGATGTTCAATATCA |
| PExPGMcy RP | GTCGACTTATGTAATAACAGTTGG |
| SiPGMcy FP | GGGGACAAGTTTGTACAAAAAAGCAGGCTCTGATACGGCAGGAGTGGAC |
| SiPGMcy RP | GGGGACCACTTTGTACAAGAAAGCTGGGTATGGTGATGTTCAATATCAC |
| PVYRT FP | ACGTCCAAAATGAGAATGCC |
| PVRRT RP | TTCAATGCACCAAACCATAA |
| qPGMcy FP | GTTAACAAGATTATAAAGGGA |
| qPGMcy RP | CCTGAGAATCTCTTCCAATC |
| CPEX FP | GAATTCCCATGGGAAATGACACAATCG |
| CPEX RP | GTCGACTCACATGTTCTTGACTCCAAGTAG |
